# A Pilot Study on Characteristics of Metabolomics and Lipidomics according to Sasang Constitution

**DOI:** 10.1155/2018/9214960

**Published:** 2018-05-30

**Authors:** Min Jung Kim, Da-Hye Lee, Jiyun Ahn, Tae-Youl Ha, Young Jin Jang, Eunju Do, Chang Hwa Jung

**Affiliations:** ^1^Division of Nutrition and Metabolism Research, Korea Food Research Institute, Seongnam, Republic of Korea; ^2^Department of Food Biotechnology, Korea University of Science and Technology, Seongnam, Republic of Korea; ^3^Clinical Trial Convergence Commercialization Team, Korean Medicine Industry Support Center, Daegu Technopark, Suseong-gu, Daegu, Republic of Korea

## Abstract

Although classification of an individual's Sasang constitution is a key step in the prescription of traditional Korean medicine, the classifying process is complex and not objective. Identification of metabolic-based biomarkers could allow the development of a reliable and sensitive classification technique and even therapeutic management. Our pilot study investigated whether metabolites in plasma are characteristic of Sasang constitutions. Ultra-high-performance liquid chromatography-quadrupole time-of-flight mass spectrometry-based metabolic analysis was conducted against 15 Soyangin (SY), 15 Taeeumin (TE), and 18 Soeumin (SE) individuals, as classified according to the Questionnaire for Sasang Constitution Classification II (QSCC II) and specialist diagnosis. Metabolomics data showed that the TE group was significantly separated from the SY and SE groups. Nine canonical pathways related to constitution; phenylalanine metabolism, aminoacyl-tRNA, tyrosine, and tryptophan biosynthesis were activated in the TE group as compared with the other groups. Similar to the results of the metabolomics analysis, the TE group was also significantly separated from the other two groups by lipidomic analysis. On the other hand, the intensity of lipid metabolites was higher in the SY group than in the other groups. Our findings suggest that the combined analysis of metabolomics and lipidomics can provide useful information for characteristics of Sasang constitutions.

## 1. Introduction

Since the results of the Human Genome Project were made known in June 2000, the promise of personalized medicine has been actively pursued; however, this remains in its infancy and there is consensus that further research is needed before personalized medicine becomes an actuality. Although oriental medicine cannot be regarded as personalized medicine, it can be regarded as a group-customized medicine, because it does not treat a condition in the same way across the board, but treatments differ according to the constitutional group to which the patient belongs [1]. Oriental constitutional medicine provides a way to balance human body and mind according to the constitution, not only for the treatment of diseases but also for healthy living [2].

Constitutional medicine in Korea is called Sasang. Sasang constitutional medicine was initiated by Jema Lee (1837–1900) and is the most representative traditional medicine of Korea. It uses an approach of considering how diseases progress differently among different constitutional groups of people and administering treatments based on the recognition of distinct patterns present in each constitutional type [3]. The constitution is classified into four types according to individual's physical and psychological characteristics [4]: Taeyangin (TY), Soyangin (SY), Taeeumin (TE), and Soeumin (SE) [2]. Traditional Korean medicine doctors claim that determination of the constitutional types could be valuable for the prevention and treatment of diseases, because disease susceptibilities and drug responses vary depending on the individual's constitution [5], although scientific evidence is still required to verify this claim.

Although the scientific basis of constitutional medicine is still in question, constitution-based oriental medicine is recognized as a medical practice in Korea. However, the Sasang constitution has the disadvantage that it is difficult to diagnose objectively, as the diagnosis of the constitution is made according to the subjective view of the oriental medicine doctor. If a scientific validation method could be developed which allows easy determination of the constitution, it will be highly useful in oriental medicine. Numerous attempts have been made to classify the constitution scientifically; the Questionnaire for Sasang Constitution Classification II (QSCC II) has recently been developed and has been used as a validated, objective assessment [6], although it is continuously being supplemented with additional surveys to improve the accuracy of determination [7]. Questionnaires about the face, body, shape, and personal characteristics have been used in trials of Sasang constitutional diagnosis [8]. Moreover, discrimination of the Sasang constitution, depending on the characteristics of the skin, fingerprints, and voice, has been reported [9–11].

In metabolomics, the causes of life phenomena are systematically identified by analyzing the composition and concentration of metabolic groups that change under various genetic, physiological, or environmental conditions [12]. The emerging field of “metabolomics,” in which a large number of small molecules from body fluids or tissues are detected quantitatively in a single step, holds immense potential for early diagnosis, identification of novel drug targets, customized drug treatment, therapy monitoring, and advances in the understanding of the pathogenesis of many diseases [13]. To date, no study has reported the relationship between Sasang constitution and metabolites.

Our study investigated whether blood metabolites differ significantly depending on the Sasang constitution as classified by QSCCII diagnosis. Polar metabolites and lipids were identified using liquid chromatography-mass spectrometry- (LC-MS-) based metabolomics and lipidomics. The analysis was performed on only three of the Sasang constitutions, as the TY constitution is very rare.

## 2. Materials And Methods

### 2.1. Subjects and Study Design

This human study was approved (KMISC-FD-22) by Daegu Oriental Hospital of Daegu Haany University (Daegu, South Korea). Healthy Korean males between 21 and 29 years of age were recruited. Each participant was grouped according to Sasang constitutional types (SCT), using the integrated diagnosis model combining four individual models based on Questionnaire for Sasang Constitutional Classification II (QSCC II), face, body shape, and voice, by oriental medicine doctors (Daegu Haany University Medical Center). Participants with a body mass index (BMI) < 18.5 kg/m^2^ or > 30 kg/m^2^ [14], hypertension, and ALT and AST greater than twice the normal upper limit or who were participating in any other study or had donated blood during the last 1 month before the start of this study or had preexisting chronic diseases or were taking drugs were excluded. Fifteen TE, 15 SY, and 18 SE individuals were identified. The TY type is extremely rare in the population [15], and no such subjects were classified in our study. The subjects had fasted 10–17 h prior to collection of blood samples in an EDTA plasma tube. Blood samples were centrifuged at 1550 ×* g* for 10 min at 4°C to collect the plasma. General characteristics of the participants, such as BMI, waist-to-hip ratio (WHR), visceral fat area (VFA), blood pressure, and plasma lipid profiles, were determined.

### 2.2. Metabolomics Analysis

Plasma protein was precipitated by addition of cold acetonitrile. After shaking for 30 min at 4°C, the samples were centrifuged at 9,000 × g for 10 min at 4°C and the supernatant was dissolved in 20% aqueous methanol containing caffeine [16]. The analyses were performed using an ACQUITY Ultra-Performance Liquid Chromatography (UPLC) system (Waters, Milford, MA, USA) coupled to an ultra-performance liquid chromatography-quadrupole time of flight mass spectrometry (UPLC-ESI/Q-TOF) system (Waters Corp., Manchester, UK) with ACQUITY UPLC BEH C18 (2.1 × 100 mm, 1.7 *μ*m) column. The column temperature was conditioned at 30°C. The flow rate was set at 0.30 *μ*l/min. The mobile phase was composed of (A) 0.1% formic acid aqueous solution and (B) 0.1% formic acid in acetonitrile, at an injection volume of 5 *μ*l. The conditions were as follows: initial condition of 98% A, 0–13 min with 98–5% A, and 13–14 min with 5% A and returning to 98% A for a 2 min reequilibration step. The mass spectrometer was a Waters SYNAPT G2-Si mass spectrometer with an electrospray ionization (ESI) mode. The ESI source was set in positive and negative ESI mode with a scan range of m/z 50–1,000. Argon was used as collision gas, and nitrogen was used as desolvation gas. The voltage of capillary, cone, and collision energy was set at 3.0 kV, 40 V, and 25 V, respectively. The gas flow for desolvation and cone was 800 and 50 L/h. The source temperature and desolvation gas temperature were 110 and 350°C, respectively.

### 2.3. Lipidomics Analysis

The sample extraction is briefly described as follows [17, 18]. Serum samples (50 *µ*l) were extracted with a mixture of chloroform and methanol (1:2; 200 *µ*l). This solution was then vortexed for 30 min at room temperature and immediately vortexed. The sample was centrifuged at 13,000 rpm at 4°C. The supernatant was completely evaporated under a stream of nitrogen. The lipid extract was diluted with an isopropanol : acetonitrile : water (2:1:1, v/v/v) solution. Lipid extracts were analyzed on a SYNAPT G2-Si mass spectrometer (Waters, Manchester, UK) coupled to an ACQUITY UPLC system (Waters, Milford, MA, USA) with an ACQUITY UPLC CSH C18 (2.1 × 100 mm, 1.7 *μ*m) column. The column temperature was set at 55°C. Mobile phase A was 10 mM ammonium acetate and 0.1% formic acid in an acetonitrile : water mixture (60:40, v/v); mobile phase B was 10 mM ammonium acetate and 0.1% formic acid in an isopropanol : acetonitrile mixture (90:10, v/v). The gradient profile was 60-43% B over 2 min, 50% B at 2.1 min, 54% B at 12 min, and 99% B at 18 min, followed by equilibration at 40% B for 2 min at a flow rate of 0.4 *μ*l/min. The Q-TOF-MS was operated in positive and negative electrospray ionization mode within a mass range of 100–1,000* m/z*. The source temperature was set at 120°C, voltage of the sampling cone is 30 V, and the desolvation gas (800 L/h) is at 250°C. The collision energy was ramped from 20 to 35 V and the capillary voltages were set at 2.0 kV (for positive) and 1.0 kV (for negative). All spectrum data were collected in continuum format using the MS^E^ acquisition mode. Mass accuracy was calibrated using sodium format, and leucine enkephalin was used as lock mass.

### 2.4. Data Processing And Statistical Analysis

The raw data were processed using Progenesis QI data analysis software (Nonlinear Dynamics, Newcastle, UK) for chromatographic alignment, normalization, peak picking, and compound identification. The resulting data sets were imported into SIMCA-P version 12.0.1 (Umetrics, Umeå, Sweden) for multivariate analysis and were mean-centered scaled. Assignment of metabolites contributing to the observed variance was performed using ChemSpider (http://www.chemspider.com), Human Metabolome Database (http://www.hmdb.ca), and LIPID MAPS (http://www.lipidmaps.org). Metabolic pathway analysis was performed using MetaboAnalyst 3.0 (http://www.metaboanalyst.ca), which is based on database sources such as KEGG and HMDB, to identify the metabolic pathways that are affected and to facilitate biological interpretation. One-way analysis for variance (ANOVA) with Tukey's post hoc tests was carried out for the comparison between three groups. Potential biomarkers were selected using VIP > 1 and* P *< 0.05.

## 3. Results

### 3.1. Subjects' Characteristics

Table 1 shows the general characteristics of participants. There were significant differences between the three groups in terms of BMI, body fat percentage (BFP), WHR, VFA, and systolic blood pressure (SBP). Although there was no significant difference between the SY and TE groups, both groups were significantly different from the SE group. SE persons have a more skinny body phenotype than SY and TE persons. This result is consistent with the results of previous studies showing a relatively low BMI for SE people [19]. In addition, total cholesterol (TC) was higher in the TE group than in the SY group; however, there was no significant difference when compared to the SE group. SBP levels were higher in the TE and SY groups than in the SE group. It is known that there may be constitutions among the Sasang constitutions that are likely to become obese and that there are differences in constitutional characteristics according to lifestyle.

### 3.2. Metabolic Profiles of Plasma Samples

**Figure 1:**
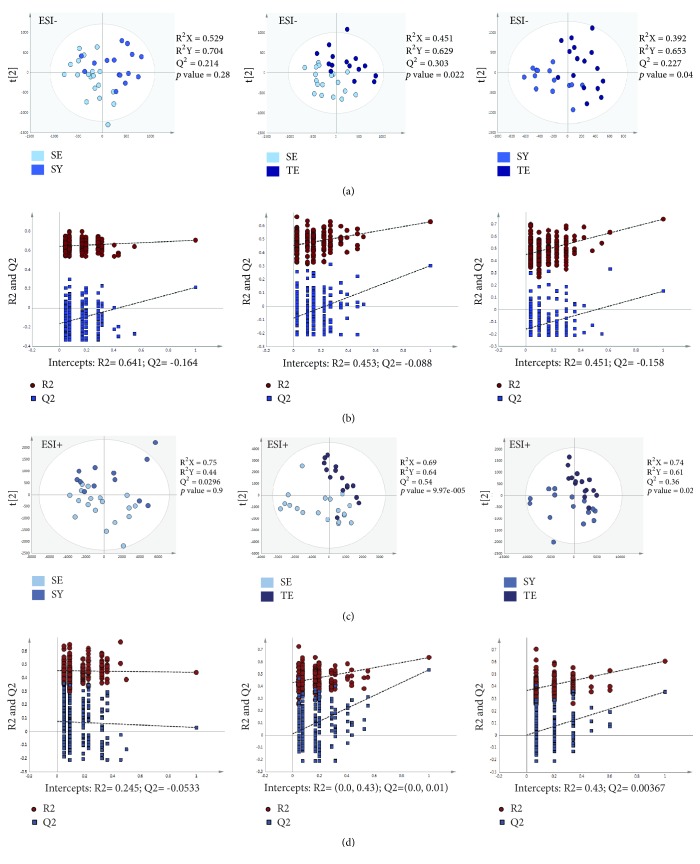
Partial least-squares discriminant analysis (PLS-DA) score and permutation results from plasma metabolites of participants classified according to Sasang constitution types based on metabolomics analysis. (a) PLS-DA score plot in ESI- mode. (b) Validation plot in ESI- mode. (c) PLS-DA score plot in ESI+ mode. (d) Validation plot in ESI+ mode. SY: Soyangin; TE: Taeeumin; SE: Soeumin.

The metabolomic profiles of plasma samples from the three groups (15 TE, 15 SY, and 18 SE) were characterized and compared. A total of 3,000 molecular features in positive and negative modes were obtained and subjected to statistical analysis using Progenesis Q1 and SIMCA-P version 12.0.1 software, including partial least-squares discriminant analysis (PLS-DA). As shown in the PLS-DA score plots, the TE group was significantly separated from the other two groups (Figures 1(a) and 1(c)). On the other hand, there was no significant difference between the SY and SE groups. The PLS-DA score plots between the Sasang constitutions showed a similar pattern in both negative and positive ion modes. The validation plots from the permutation tests strongly supported the validity of the PLS-DA scores (Figures 1(b) and 1(d)). Validity was supported by the finding that all permuted R^2^ and Q^2^ values on the left were lower than the original point on the right and that the Q^2^ regression line (in blue in the plots in Figure 1) had a negative intercept.

A total of 33 metabolites (VIP > 1,* P* < 0.05) were identified as potential biomarkers for diagnosis of Sasang constitution types (Table 2). Among the 33 metabolites, the intensity of 27 metabolites showed the same tendency in order of TE, SE, and SY groups. In a comparison of the TE and SY groups, a total of 23 metabolites, such as 3-hydroxybutyric acid, leucine, caffeic acid, melatonin, and phenyllactic acid, were significantly higher in the TE than in the SY types. In addition, the levels of 4-hydroxybenzaldehyde, glutamic acid, leucine, indole-3-lactic acid, acetylcarnitine, melatonin, and stearoylcarnitine were significantly different between the TE and SE groups. Eight significant metabolites were identified in the comparison of the SY and SE groups. In particular, glutamic acid may be a potential biomarker candidate for the diagnosis of Sasang constitution type. The relative intensity of each metabolite in the sample from each group was shown in heat map (Figure 3(a)) MetaboAnalyst indicates that 11 pathways, such as phenylalanine metabolism; aminoacyl-tRNA biosynthesis; tyrosine and tryptophan biosynthesis; D-glutamine and D-glutamate metabolism; valine, leucine, and isoleucine biosynthesis; and nitrogen metabolism, were regarded as the targeted metabolic pathways (Figure 3(b)). This result suggests that the difference in Sasang constitutions mainly involves differences in amino-acid-related metabolism.

### 3.3. Lipidomic Profiles of Plasma Samples

**Figure 2:**
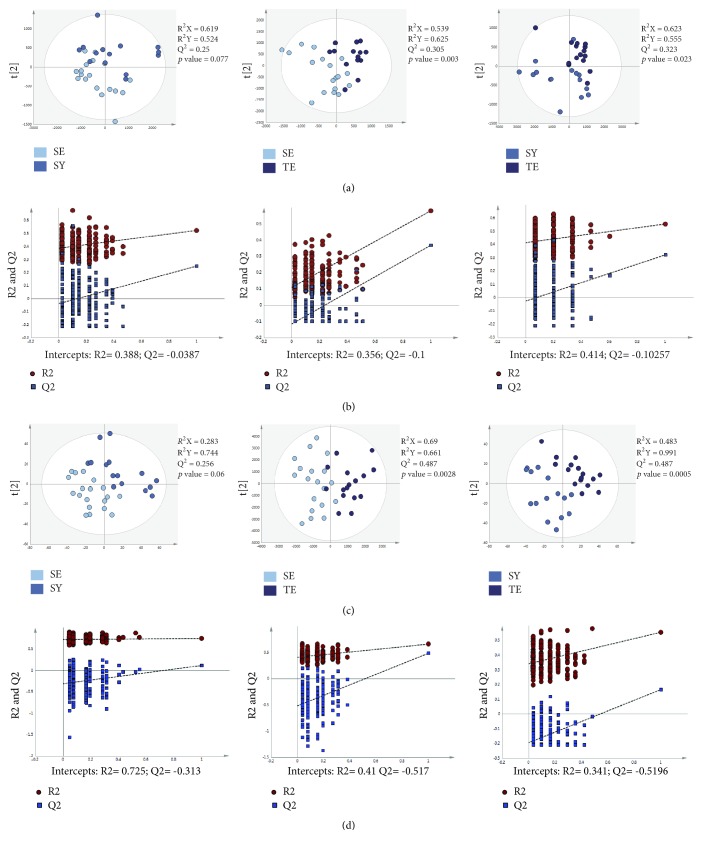
Partial least-squares discriminant analysis (PLS-DA) score and permutation results from plasma metabolites of participants classified according to Sasang constitution types using lipidomics analysis. (a) PLS-DA score plot in ESI- mode. (b) Validation plot in ESI- mode. (c) PLS-DA score plot in ESI+ mode. (d) Validation plot in ESI+ mode. SY: Soyangin; TE: Taeeumin; SE: Soeumin.

**Figure 3:**
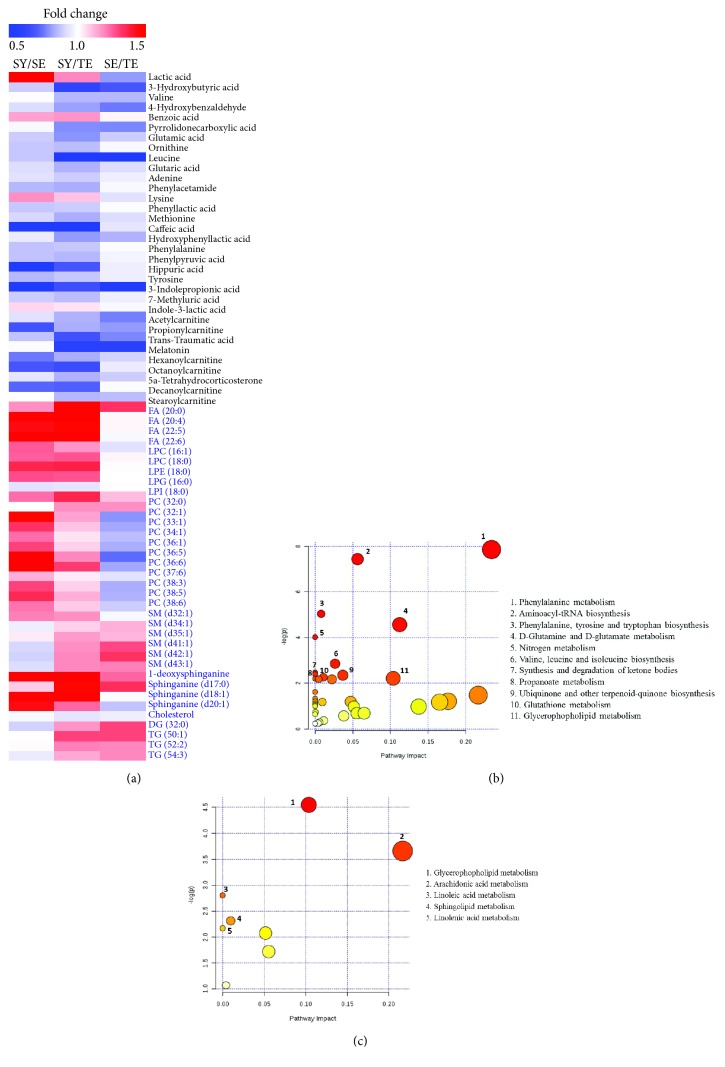
Heat map and metabolic pathway of metabolites identified by metabolomics and lipidomics analysis. (a) Heat map of identified metabolites. (b) Amino acid metabolism. (c) Lipid metabolism. SY: Soyangin; TE: Taeeumin; SE: Soeumin.

SE persons are characterized by having a slender body shape, unlike TE and SY persons [5]. Lipid profiling of individuals with these different constitutions was expected to show a clear trend between the SE and the other two constitutions. The PLS-DA score plots showed a significant separation between the TE and the other two constitutions in both positive and negative modes (Figures 2(a) and 2(c)). The validation tests supported the validity of the PLS-DA models (Figures 2(b) and 2(d)). Although there was no significant difference in the lipid profiles of the SE and SY groups, the significance in the negative and positive modes was 0.077 and 0.06, respectively. Moreover, we identified 36 lipids (Table 3). Among the 36 metabolites, 16 metabolites, such as FA (20:4), FA (22:5), FA (22:6), LPC (16:1), LPE (16:0), LPI (18:0), PC (32:1), PC (33:1), PC (34:1), 1-deoxysphinganine, sphinganine (d18:1), and sphinganine (d20:1), were significantly grouped among the three groups. In the comparison of the SY and SE groups, 20 fatty acids showed significant differences, which was more than the eight metabolites showing differences in the metabolomics profiles. Our results showed that the lipidomic analysis showed that the SY group was characterized by different lipid profiles as compared with SE group (Figure 3(a)). Interestingly, MetaboAnalyst analysis showed that there were five canonical pathways that may be related to Sasang constitutions (Figure 3(c)); the top two pathways were glycerophospholipid and arachidonic acid metabolism. In the SY group, these metabolic pathways were more activated than in the SE and TE groups.

## 4. Discussion

This study investigated whether metabolomics and lipidomics analyses of human plasma could classify the Sasang constitution types. Sasang constitutional medicine prescribes medicinal herbs according to the individual type, based on the biopsychosocial perspectives [5]. In terms of personalized medicine, which is a current focus point, it is surprising that such an analysis has not yet been reported. In an effort to develop a method for classifying Sasang constitution type, QSCC II method was developed [8], which could assist practitioners in diagnosing the Sasang constitution type but was limited in terms of scientific evidence [6, 20]. The accurate classification of Sasang constitution type has been a major concern in both conventional and complementary medicine and could play an important role in oriental medical prescriptions and treatment for certain diseases, such as stroke, diabetes, and metabolic syndrome [15, 21–23].

In this study, we first investigated the use of metabolomics for classifying the Sasang constitution types, as metabolic analysis has the advantage of allowing the prediction or diagnosis of human diseases, such as cancer, diabetes, and chronic liver disease [13, 24–26]. Our results showed the possibility of classifying Sasang constitution types by analyzing both metabolomics and lipidomics. The TE group was significantly separated from the SE and SY groups by metabolomics analysis, although it was not possible to distinguish between SY and SE groups. We also applied global lipid profiling to plasma to further clarify the metabolic differences between each constitution type. The results were similar to the amino-acid-related metabolites; interestingly, the lipid profiles showed the possibility to distinguish the difference between the SE and SY groups (*p* = 0.06); thus, with a larger scale study, it may be possible to distinguish these two constitutions.

It has been reported that TE persons have a high prevalence rate of obesity due to hypoactivity of their energy metabolism [3, 22, 27]. The BMI of TE group was similar to that of the SY group, but the amino acids and lipid metabolites showed a significant difference between the TE and SY groups. Although the total cholesterol levels of the TE group were significantly higher than that of the SY group, they were within the normal range. The branched-chain amino acids, that is, lysine, tryptophan, cysteine, and glutamate, have been found to be present at high levels in obese individuals [28]. In our study, valine, glutamate, leucine, and lysine were more prevalent in the TE group than in the SE and SY groups, suggesting that metabolomics analysis can provide a scientific basis for explaining the proclivity of TE people to progress to obesity. Lysine and glutamic acid levels were also higher in the TE group. In particular, glutamic acid levels were significantly different among the three groups. Among the four Sasang types, TE persons had significantly higher total cholesterol and triglyceride (TG) values in the blood than other constitutions [3]. TE persons are more likely to be obese than other constitutions; however, our results showed that the BMI in the TE group was significantly different from that of the SE group but was not different from that of the SY group.

The concentration of lipid metabolites of the SY group was greater than that of the TE group. Levels of glycerophospholipids, lysolipids, glycerolipids, and shphingolipids differed significantly between each Sasang type, and levels were particularly higher in the SY group than in the TE groups. Low lipid concentrations may represent hypoactivity of lipid metabolism, and it is presumed that TE persons are more likely to be obese than an SY person with the same BMI; BMI is used to define obesity. Among the identified biomarkers, we found some interesting and compelling results: the level of 1-deoxysphinganine in the SY group was much higher than that in the other two constitution types. These levels are also elevated in diabetic patients and have been suggested to represent an early biomarker [29–31]. TE individuals have higher prevalence of diabetes than the other two types [32]. Since 1-deoxysphinganine is a toxic lipid for insulin-producing pancreatic *β* cells [29], people with lower concentrations of the 1-deoxysphinganine can be more sensitive to this substance. In some circumstances, an increase in this substance in TE people may increase the incidence of diabetes mellitus rather than its incidence in SY people.

This study had some limitations. First, it involved a small sample size, which was inadequate to confirm the accuracy of the classification of the Sasang constitution type. Therefore, current evidence of metabolomics/lipidomics-based Sasang constitution medicine is insufficient to recommend classification according to constitution type. Second, the correlation between constitution and metabolite profiles in this study was based on healthy individuals in their twenties. So, it is not clear at this stage whether the metabolite profiles will hold across different age groups and health/disease conditions. In addition, since this study focused on male participants, further research is needed to identify gender differences in metabolites. One more limitation of this study is that it did not consider the environmental factors such as eating habit and lifestyle which might affect the results.

Collectively, the profiles of blood metabolites in TE and SY constitution types differ. Even when such individuals have the same BMI, it may thus be possible to distinguish between the two constitution types through metabolite analysis. Although this was a pilot study, the data revealed the possibility of diagnosis of the Sasang constitution type based on metabolomics and lipidomics analysis. Further clinical trials are required to verify these findings.

## Figures and Tables

**Table 1:** General characteristics of participants.

Variable	SY	TE	SE	*P* value
No. of participants	15	15	18	
Age (year)	24.07 ± 2.71	24.33 ± 2.02	23.67 ± 1.68	0.670
BMI (kg/m^2^)	25.09 ±2.21^b^	25.57 ± 1.90^b^	21.36 ± 1.75^a^	<0.0001^*∗∗∗∗*^
BFP (%)	22.65 ± 4.34^b^	24.02 ± 4.06^b^	19.08 ± 4.79^a^	0.007^*∗∗*^
WHR	0.87 ± 0.03^b^	0.86 ± 0.04^b^	0.83 ± 0.03^a^	0.003^*∗∗*^
VFA	81.91 ± 28.34^b^	86.55 ± 19.62^b^	60.54 ± 25.64^a^	0.009^*∗*^
Glucose (mg/dL)	92.20 ± 7.59	90.20 ± 4.04	91.61 ± 9.10	0.747
TC (mg/dL)	161.20 ± 32.74^a^	186.60 ± 26.28^b^	168.67 ± 27.47^ab^	0.060
TG (mg/dL)	126.87 ± 138.15	111.33 ± 65.40	74.22 ± 37.86	0.220
SGOT	22.73 ± 6.72^ab^	26.60 ± 11.63^b^	19.50 ± 4.42^a^	0.048^*∗*^
SGPT	33.87 ± 11.69	29.80 ± 13.21	24.39 ± 9.57	0.069
*γ*-GPT	32.00 ± 11.59	28.40 ± 13.28	26.17 ± 10.10	0.363
Protein (g/dL)	7.51 ± 0.26	7.57 ± 0.27	7.36 ± 0.30	0.090
Albumin (g/dL)	4.67 ± 0.23	4.77 ± 0.24	4.76 ± 0.29	0.532
SBP (mmHg)	127.40 ± 9.93^b^	126.27 ± 11.51^b^	117.56 ± 10.18^a^	0.018^*∗*^
DBP (mmHg)	81.40 ± 8.77	81.47 ± 8.59	77.94 ± 7.77	0.382
WBC (×10^3^/*μ*L)	6.03 ± 1.46	5.82 ± 1.32	5.94 ± 1.55	0.922
RBC (×10^6^/*μ*L)	4.94 ± 0.43	5.11 ± 0.35	4.82 ± 0.40	0.121
PLT (×10^3^/*μ*L)	261.87 ± 54.80	243.73 ± 43.89	235.17 ± 42.61	0.271

SY: Soyangin; TE: Taeeumin; SE: Soeumin; BMI: body mass index; BFP: body-fat percentage; WHR: waist-to-hip ratio; VFA: visceral fat area; TC: total cholesterol; TG: triglycerides; SGOT: serum glutamic oxaloacetic transaminase; SGPT: serum glutamic-pyruvic transaminase; *γ*-GPT: gamma-glutamyl transferase; SBP: systolic blood pressure; DBP: diastolic blood pressure; WBC: white blood cell; RBC: red blood cell; PLT: platelet cell. Differences among groups were analyzed using Duncan's multiple range test (*p* < 0.05) and are indicated by different lowercase letters (a, b, and ab). Letter “a” is significant to “b,” but “ab” is not significant. *∗*, *∗∗*, and *∗∗∗∗* denote one-way ANOVA (parametric test).

**Table 2:** List of potential plasma biomarkers in three Sasang constitutions.

No.	Tentative metabolites	m/z	Mass error (mDa)	Adduct	Fold change			*p* value	Pathway
					SY/SE	SY/TE	SE/TE		
1	Lactic acid	89.0235		M-H	1.49^*∗*^	1.19	0.80	0.090	Propanoate metabolism
2	3-Hydroxybutyric acid	103.0388	-0.69	M-H	0.89	0.61^*∗*^	0.68	0.020	Synthesis and degradation of ketone bodies
3	Valine	118.0737	-13.13	M+H	1.01	0.85	0.85	0.076	Aminoacyl-tRNA biosynthesis Valine, leucine, and isoleucine biosynthesis
4	4-Hydroxybenzaldehyde	121.0285	-0.53	M-H	0.92	0.81	0.75^*∗∗*^	0.041	Phenylalanine metabolism
5	Benzoic acid	123.0437	-0.94	M+H	1.15	1.17^*∗*^	1.02	0.115	Phenylalanine metabolism
6	Pyrrolidonecarboxylic acid	128.0341	-0.73	M-H	0.98	0.78	0.77	0.068	D-glutamine and D-glutamate metabolism
7	Glutamic acid	130.0376	-12.79	M+H-H_2_O	0.89^*∗*^	0.79^*∗∗∗*^	0.89^*∗*^	0.000	Aminoacyl-tRNA biosynthesis D-Glutamine and D-glutamate metabolism
8	Ornithine	131.0811	-0.96	M-H	0.88	0.86^*∗*^	0.98	0.071	Glutathione metabolism
9	Leucine	132.0883	-14.17	M+H	0.88	0.45^*∗∗*^	0.40^*∗∗*^	0.002	Aminoacyl-tRNA biosynthesis Valine, leucine, and isoleucine biosynthesis
10	Glutaric acid	133.0562	6.11	M+H	0.92	0.84^*∗*^	0.92	0.045	Lysine degradation Fatty acid metabolism
11	Adenine	136.0622	-0.12	M+H	0.93	0.89^*∗*^	0.96	0.094	Purine metabolism
12	Phenylacetamide	136.0632	-7.00	M+H	0.85	0.83^*∗*^	0.98	0.084	Phenylalanine metabolism
13	Lysine	145.0953	-2.37	M-H	1.18	1.10	0.93	0.105	Aminoacyl-tRNA biosynthesis
14	Phenyllactic acid	147.0440	-0.63	M-H_2_O-H	0.88^*∗*^	0.89^*∗*^	0.99	0.038	Phenylalanine and tyrosine metabolism
15	Methionine	148.0421	-1.10	M-H	0.91	0.83^*∗*^	0.92	0.265	Aminoacyl-tRNA biosynthesis
16	Caffeic acid	163.0296	-9.86	M+H-H_2_O	0.38^*∗∗∗*^	0.40^*∗∗∗*^	0.94	0.000	Phenylpropanoid biosynthesis
17	Hydroxyphenyllactic acid	163.0390	-0.51	M-H_2_O-H	0.95	0.80^*∗*^	0.84	0.157	Ubiquinone and other terpenoid-quinone biosynthesis
18	Phenylalanine	164.0701	-1.08	M-H	0.87^*∗*^	0.88^*∗*^	0.99	0.019	Phenylalanine metabolism Aminoacyl-tRNA biosynthesis Phenylalanine, tyrosine, and tryptophan biosynthesis
19	Phenylpyruvic acid	165.0420	-13.20	M+H	0.87	0.85^*∗*^	0.97	0.094	Phenylalanine metabolism Phenylalanine, tyrosine, and tryptophan biosynthesis
20	Hippuric acid	178.0494	-3.80	M-H	0.47^*∗*^	0.68^*∗*^	0.96	0.045	Phenylalanine metabolism
21	Tyrosine	180.0652	-0.93	M-H	0.85^*∗*^	0.88^*∗*^	0.96	0.045	Phenylalanine metabolism Aminoacyl-tRNA biosynthesis Phenylalanine, tyrosine, and tryptophan biosynthesis
22	3-Indolepropionic acid	190.0737	-7.10	M+H	0.36	0.66	0.54	0.247	
23	7-Methyluric acid	203.0191	1.05	M+Na-2H	0.89	0.86^*∗*^	0.96	0.024	Caffeine metabolism
24	Indole-3-lactic acid	204.0640	-2.15	M-H	1.07	1.05^*∗∗*^	0.98^*∗*^	0.07	Tryptophan metabolism
25	Acetylcarnitine	204.1108	-12.79	M+H	0.93	0.84^*∗*^	0.76^*∗*^	0.017	Fatty acid metabolism (acyl carnitine)
26	Propionylcarnitine	218.1265	-12.67	M+H	0.67^*∗*^	0.83	0.80	0.033	Fatty acid metabolism (acyl carnitine)
27	*trans*-Traumatic acid	227.1272	-1.14	M-H	0.87	0.67^*∗*^	0.77	0.053	
28	Melatonin	231.1222	8.82	M-H	0.99	0.58^*∗*^	0.59^*∗∗*^	0.063	Tryptophan metabolism
29	Hexanoylcarnitine	260.1748	-11.40	M+H	0.75^*∗*^	0.83	0.90	0.042	Fatty acid metabolism (acyl carnitine)
30	Octanoylcarnitine	288.2072	-10.32	M+H	0.68	0.65^*∗*^	0.95	0.033	Fatty acid metabolism (acyl carnitine)
31	5a-Tetrahydrocorticosterone	315.2278	-4.6	M+H-2H_2_O	0.94	0.84^*∗*^	0.89	0.090	
32	Decanoylcarnitine	316.2402	-8.55	M+H	0.72	0.71^*∗*^	0.99	0.071	Fatty acid metabolism (acyl carnitine)
33	Stearoylcarnitine	428.3683	2.29	M+H	1.00	0.85^*∗*^	0.86^*∗*^	0.047	Fatty acid metabolism (acyl carnitine)

SY: Soyangin; TE: Taeeumin; SE: Soeumin. Fold change was calculated by dividing the mean of the peak intensity of each metabolite from each of the two groups. ^*∗*^*p* < 0.05; ^*∗∗*^*P* < 0.01; ^*∗∗∗*^*P* < 0.001.

**Table 3:** Circulating lipid profiles in the three Sasang constitutions.

No.	Identity	m/z	Mass error (mDa)	Adduct	MS fragments (ESI)	Fold change			*p* value	Pathway
						SY/SE	SY/TE	SE/TE		
1	FA (20:0)	311.2904	-4.6	M-H	183	1.18	1.58^*∗*^	1.33	0.116	Lipid metabolism
2	FA (20:4)	303.2241	-8.3	M-H	183	1.50^*∗*^	1.53^*∗*^	1.02	0.049	Arachidonic acid metabolism
3	FA (22:5)	329.2444	-3.7	M-H	183	1.44^*∗*^	1.47^*∗*^	1.02	0.029	Lipid metabolism
4	FA (22:6)	327.2340	1.6	M-H	183	1.88^*∗*^	1.84^*∗*^	0.98	0.005	Lipid metabolism
5	LPC (16:1)	494.3254	0.72	M+H	476, 184, 104	1.26^*∗*^	1.17	0.93	0.026	Lysolipid metabolism
6	LPC (18:0)	568.3622	1.1	M+COOH	508, 283, 183, 153	1.25	1.27^*∗*^	1.02	0.064	Lysolipid metabolism
7	LPE (16:0)	452.2772	-2.00	M-H	255, 214, 196	1.36^*∗*^	1.37^*∗*^	1.01	0.015	Lysolipid metabolism
8	LPE (18:0)	480.3096	5.2	M-H	283, 214, 196	1.28^*∗*^	1.27	1.00	0.054	Lysolipid metabolism
9	LPG (16:0)	507.2762	1.1	M+Na	449, 311, 155	0.93^*∗*^	0.94	1.01	0.088	Lysolipid metabolism
10	LPI (18:0)	599.3170	-2.6	M-H	315, 283, 241	1.23	1.36^*∗*^	1.11	0.044	Lysolipid metabolism
11	PC (32:0)	734.5695	-0.50	M+H	184	1.00	1.18^*∗*^	1.18^*∗*^	0.061	Glycerophospholipid metabolism
12	PC (32:1)	732.5528	1.68	M+H	184	1.46^*∗*^	1.15	0.79^*∗*^	0.040	Glycerophospholipid metabolism
13	PC (33:1)	746.5676	-2.38	M+H	184	1.33^*∗*^	1.10	0.82^*∗*^	0.048	Glycerophospholipid metabolism
14	PC (34:1)	760.5878	2.22	M+H	184	1.23^*∗*^	1.05	0.86	0.033	Glycerophospholipid metabolism
15	PC (36:1)	788.6171	2.97	M+H	184	1.30^*∗∗*^	1.08	0.83	0.070	Glycerophospholipid metabolism
16	PC (36:5)	780.5535	-0.81	M+H	184	1.63^*∗*^	1.19	0.73^*∗*^	0.072	Glycerophospholipid metabolism
17	PC (36:6)	778.5426	3.94	M+H	184	1.59^*∗∗*^	1.31	0.82	0.005	Glycerophospholipid metabolism
18	PC (37:6)	792.5567	2.40	M+H	184	1.13^*∗*^	1.04	0.93^*∗*^	0.041	Glycerophospholipid metabolism
19	PC (38:3)	812.6167	-0.18	M+H	184	1.30^*∗*^	1.08	0.83	0.055	Glycerophospholipid metabolism
20	PC (38:5)	808.5840	-1.64	M+H	184	1.35^*∗*^	1.13	0.84	0.084	Glycerophospholipid metabolism
21	PC (38:6)	806.5732	2.44	M+H	184	1.22^*∗*^	1.09	0.89	0.063	Glycerophospholipid metabolism
22	SM (d32:1)	675.5440	-0.13	M+H	184	1.20^*∗*^	1.18	0.98	0.036	Sphingolipid metabolism
23	SM (d34:1)	703.5772	2.37	M+H	184	0.96	1.08	1.13^*∗*^	0.117	Sphingolipid metabolism
24	SM (d35:1)	717.5886	-2.40	M+H	184	1.04	1.16^*∗*^	1.12	0.057	Sphingolipid metabolism
25	SM (d41:1)	801.6847	-0.30	M+H	184	0.91	1.18	1.29^*∗∗*^	0.052	Sphingolipid metabolism
26	SM (d42:1)	815.7003	-0.30	M+H	184	0.90	1.19	1.33^*∗∗*^	0.052	Sphingolipid metabolism
27	SM (d43:1)	829.7133	-3.05	M+H	184	0.92	1.19	1.20^*∗∗*^	0.052	Sphingolipid metabolism
28	1-Deoxysphinganine	286.3099	-0.70	M+H	270	3.59^*∗*^	4.45^*∗*^	1.24	0.009	Sphingolipid metabolism
29	Sphinganine (d17:0)	288.2895	-3.53	M+H	282, 264	1.09	1.46	1.34^*∗∗*^	0.197	Sphingolipid metabolism
30	Sphinganine (d18:1)	300.2907	-2.44	M+H	310	1.93	1.91^*∗*^	0.99	0.034	Sphingolipid metabolism
31	Sphinganine (d20:1)	310.3120	-0.25	M+H	268	1.44^*∗*^	1.24	0.87	0.040	Sphingolipid metabolism
32	Cholesterol	369.3534	-0.17	M+H	369	0.98	0.94^*∗*^	0.96	0.061	Primary bile acid biosynthesis
33	DG (32:0)	591.4954	-3.40	M+Na	551, 313, 257	0.90	1.17	1.30^*∗∗*^	0.012	Glycerolipid metabolism
34	TG (50:1)	850.7851	-1.82	M+NH_4_	603, 551	1.00	1.30	1.30^*∗∗*^	0.472	Glycerolipid metabolism
35	TG (52:2)	876.8014	4.19	M+NH_4_	859, 841, 577	1.01	1.20	1.19^*∗∗*^	0.591	Glycerolipid metabolism
36	TG (54:3)	902.8162	-2.55	M+NH_4_	867, 603, 339	0.95	1.14	1.19^*∗∗∗*^	0.346	Glycerolipid metabolism

Fold change was calculated by dividing the mean of the peak intensity of each metabolite from each of the two groups. SY: Soyangin; TE: Taeeumin; SE: Soeumin. ^*∗*^*p* value < 0.05.

## Abbreviations

BFP: Body fat percentage
BMI: Body mass index
PLS-DA: Partial least-squares discriminant analysis
QSCC II: Questionnaire for Sasang Constitution Classification II
SBP: Systolic blood pressure
SE: Soeumin
SY: Soyangin
TC: Total cholesterol
TE: Taeeumin
TG: Total triglyceride
VFA: Visceral fat area
WHR: Waist-to-hip ratio.
